# Efficacy of FOLFIRI plus cetuximab vs FOLFIRI plus bevacizumab in 1st-line treatment of older patients with *RAS* wild-type metastatic colorectal cancer: an analysis of the randomised trial FIRE-3

**DOI:** 10.1038/s41416-022-01854-y

**Published:** 2022-05-30

**Authors:** Laura E. Fischer, Sebastian Stintzing, Ludwig Fischer von Weikersthal, Dominik P. Modest, Thomas Decker, Alexander Kiani, Florian Kaiser, Salah-Eddin Al-Batran, Tobias Heintges, Christian Lerchenmüller, Christoph Kahl, Gernot Seipelt, Frank Kullmann, Martina Stauch, Werner Scheithauer, Clemens Giessen-Jung, Jens Uhlig, Bettina Peuser, Claudio Denzlinger, Arndt Stahler, Lena Weiss, Kathrin Heinrich, Swantje Held, Andreas Jung, Thomas Kirchner, Volker Heinemann

**Affiliations:** 1 Department of Medicine III, LMU University Hospital Munich, München, Germany; 2 Charité—Universitätsmedizin Berlin, corporate member of Freie Universität Berlin and Humboldt-Universität zu Berlin, Department of Hematology, Oncology and Cancer Immunology, Campus Mitte, Berlin, Germany; 3 Gesundheitszentrum St. Marien, Amberg, Germany; 4 Oncological Practice, Ravensburg, Germany; 5 Klinikum Bayreuth GmbH, Bayreuth, Germany; 6 Oncological Praxis, Landshut, Germany; 7 Institute of Clinical Cancer Research at Krankenhaus Nordwest University Cancer Center, Frankfurt, Germany; 8 Rheinlandklinikum Neuss, Lukaskrankenhaus, Neuss, Germany; 9 Oncological Practice, Münster, Germany; 10 Department of Hematology, Oncology and Palliative Care, Klinikum Magdeburg gGmbH, Magdeburg, Germany; 11 Department of Internal Medicine, Clinic III—Hematology, Oncology and Palliative Care, Rostock University Medical Center, Rostock, Germany; 12 Oncological Practice, Bad Soden, Germany; 13 Department of Medicine I, Klinikum Weiden, Weiden, Germany; 14 Oncological Practice, Kronach, Germany; 15 Department of Internal Medicine I & Comprehensive Cancer Center, Medical University Vienna, Vienna, Austria; 16 Oncological Practice, Naunhof, Germany; 17 Onkologische Praxis am Diakonissenhaus, Leipzig, Germany; 18 Marienhospital, Stuttgart, Germany; 19 linAssess GmbH, Leverkusen, Germany; 20 Institut für Pathologie, Ludwig-Maximilians-Universität, München, Germany; 21 Comprehensive Cancer Center (CCC Munich LMU), LMU University Hospital Munich, Munich, Germany

**Keywords:** Targeted therapies, Colon cancer, Chemotherapy

## Abstract

**Background:**

The evidence on the efficacy of anticancer therapy is limited in older patients with metastatic colorectal cancer (mCRC). This retrospective analysis of phase III FIRE-3 trial assesses the efficacy of FOLFIRI plus either cetuximab or bevacizumab according to the patients’ age and sidedness of primary tumour.

**Methods:**

The study endpoints overall response rate (ORR), progression-free survival (PFS) and overall survival (OS) were compared between younger (<65 years) and older (≥65 years) patients, followed by stratification according to primary tumour sidedness. ORR was compared using Fisher´s exact test, OS and PFS were estimated by the Kaplan–Meier method and compared using the log-rank test. Univariate Cox regression analyses assessed hazard ratios and 95% confidence intervals for OS and PFS.

**Results:**

Overall, older patients with *RAS* WT tumours had a significantly shorter OS when compared to younger patients (25.9 months vs 29.3 months, HR 1.29; *P* = 0.02). Also the proportion of right-sided tumours was significantly greater in older patients (27.1% vs 17.9%; *P* = 0.029). Secondary resection rates were numerically higher in younger patients (25.4% vs. 17.6%, *P* = 0.068) than in older patients. This was primarily seen in the Cetuximab arm, where older patients underwent less likely resection (13.1% vs. 26%; *P* = 0.02). Older patients with left-sided tumours showed only a trend towards greater efficacy of cetuximab (HR 0.86; *P* = 0.38). In patients with right-sided primary tumours, older patients did not appear to benefit from cetuximab in contrast to younger patients (≥65 years: 16.6 months vs 23.6 months, HR 1.1; *P* = 0.87; <65 years: 21.9 months vs 16.4 months HR 1.5; *P* = 0.31).

**Conclusions:**

In FIRE-3, OS was generally shorter in older patients in comparison to younger patients. This could be explained by the overrepresentation of right-sided tumours and a lower secondary resection rate in older patients. The efficacy of targeted therapy was dependent on tumour sidedness in older patients with *RAS* WT mCRC.

**Clinical trial:**

FIRE-3 (NCT00433927).

## Introduction

Worldwide, colorectal cancer (CRC) is the third most frequent cancer and the second most frequent cause of cancer-related mortality [1]. In the last decades, CRC-associated mortality has been declining in patients from highly developed European countries. This decrease was, however, less pronounced in older people [2]. Several reports support the notion that mCRC in older patients is associated with a less favourable outcome than in younger patients [3, 4]. This finding is most likely multifactorial and can be explained by higher rates of comorbidities and frailty [5] as well as different treatment regimens throughout age cohorts [6]. In addition, the molecular biology underlying mCRC may be different in older patients with regard to tumour mutational load, epigenetic modifications or telomere dysfunction [7, 8].

Despite an increasing incidence of CRC at progressing age, a conclusive definition of older patients has not been established [9]. Patients at higher ages are hardly studied in clinical trials. Therefore, evidence-based therapy for this age group remains an unsolved issue. Current guidelines recommend stratification of patients to undergo intensified treatment not only by age but also by fitness [10, 11]. This stratification is essential, as older patients might not receive further treatment beyond first-line in contrast to younger patients.

Based on results from several randomised studies, treatment options for frail patients usually include fluoropyrimidines in combination with bevacizumab (FP/bev) [12–16]. In a predominantly older patient population, the sequential escalation from FP/bev to an irinotecan-based doublet plus bevacizumab at tumour progression failed to confirm non-inferiority to upfront combination therapy but showed different efficacy patterns according to molecular subgroups and gender [17]. Doublet chemotherapy showed a manageable safety profile but no clear survival benefit for elderly patients [18].

Data regarding the efficacy of anti-*EGFR* antibodies such as cetuximab [19] in older patients are, however, limited to retrospective or small prospective studies of often molecularly unselected patients.

We therefore aimed to investigate the impact of targeted biological therapies (cetuximab or bevacizumab) in combination with doublet chemotherapy (FOLFIRI) as a first-line regimen in a retrospective subgroup analysis of older patients with *RAS* WT metastatic CRC of the randomised phase III FIRE-3 trial (AIO KRK 0306). Here we examined not only the association of age and survival endpoints but also analysed treatment efficacy in relation to primary tumour location and patient age.

## Methods

### Study design and patients

Study design, eligibility criteria and treatment parameters of FIRE-3 have previously been reported [20]. Randomisation was done centrally via fax using permuted blocks of randomly varying sizes. Stratification was according to ECOG performance status (0–1 or 2), a number of metastatic sites (1 or >1), white blood cell count (<8 × 10^9^ cells per L or ≥8 × 10^9^ cells per L) and alkaline phosphatase concentration (<300 units per L or ≥300 units per L) [20]. The study was carried out in accordance with the Declaration of Helsinki. All patients provided written informed consent prior to their participation.

The primary endpoint was the investigator-assessed objective response rate (ORR, complete or partial response) according to the Response Evaluation Criteria in Solid Tumours (RECIST) criteria, version 1.0.5. Secondary endpoints included progression-free survival (PFS) and overall survival (OS). The statistical design of FIRE-3 has been described elsewhere [20].

In the post-hoc analysis of FIRE-3, efficacy results of a subgroup of patients with tumours that were wild-type at the *RAS* genes *KRAS* and *NRAS* exons 2–4 were presented (final *RAS* wild-type [21]).

In the final survival analysis 2021, the per-protocol population was defined and analysed [22]. In the present analysis, all patients with the final *RAS* wild-type of the post-hoc analysis [21] were included. Patients were grouped into cohorts over 65 plus 65 (≥65) or under 65 (<65) for age-related analysis.

Primary tumours originating from the caecum to the transverse colon were assigned to the right colon, and tumours of the splenic flexure to the rectum to the left colon, respectively.

### Statistical analysis

Survival-based outcomes were analysed by the Kaplan–Meier method and described by median values. Comparisons of survival-based outcomes were conducted using log-rank tests and Cox regression analyses that were described as hazard ratios [17] with 95% confidence intervals (95% CI). Response rates and early tumour shrinkage were compared by Fisher’s exact test (two-sided). Differences in the depth of response between patients treated with FOLFIRI plus cetuximab and FOLFIRI plus bevacizumab were investigated with a two-sided Wilcoxon test. Where indicated, odds ratios (ORs) and hazard ratios (HRs) were calculated.

Toxicity was assessed using the NCI-CTCAE version 4.0 in all patients that received treatment within the study. Comparisons of symptomatic toxicities were conducted by Fisher’s exact test.

*P*-values <0.05 were considered statistically significant. Statistical analyses were performed using SPSS version 25 for Windows (SPSS Inc, Chicago, IL).

## Results

### Study populations and baseline characteristics

In FIRE-3, eligible patients were aged 18–75 years [20–22]. Among 400 patients with final *RAS* wild-type mCRC, 199 were treated with FOLFIRI plus cetuximab and 201 with FOLFIRI plus bevacizumab. Subdivision of the whole study population at a cut-off of 65 years yielded nearly equally sized patient cohorts. The median age of the investigated population was 64 years. The younger age cohort (<65 years) contained 201 patients (50.2%), while 199 patients (49.8%) were included in the older cohort (≥65 years).

In the overall study population, 77% of patients presented with left-sided and 22.5% with right-sided primary tumours, while in 0.5% of patients primary tumour location could not be determined. Primary tumours were left-sided in 72.4% of older (age ≥65 years) and in 81.6% of younger patients (age <65 years). As a result, the proportion of right-sided tumours was significantly greater in older patients (27.1% vs 17.9%; *P* = 0.029). Other patient- and tumour-related characteristics were balanced between age groups. For baseline characteristics according to age subgroups please refer to Table 1.

**Table 1:** Patient and tumour characteristics according to age categories and treatment arm.

Characteristic	Age analysis set			
	<65 years, *n* = 201 (50.2%)		≥65 years, *n* = 199 (49.8%)	
	FOLFIRI + cetuximab	FOLFIRI + bevacizumab	FOLFIRI + cetuximab	FOLFIRI + bevacizumab
Treatment arm, *n* (%)	100 (49.8)	101 (50.2)	99 (49.7)	100 (50.3)
Gender, *n* (%)				
Male	79 (79)	62 (61.4)	67 (67.7)	71 (71)
Female	21 (21)	39 (38.6)	32 (32.3)	29 (29)
ECOG^a^ performance status, *n* (%)				
0	60 (60)	59 (58.4)	46 (46.5)	50 (50)
1	39 (39)	40 (39.6)	51 (51.5)	49 (49)
2	1 (1)	2 (2)	2 (2.0)	1 (1)
BRAF status, *n* (%)				
RAS wt/BRAF wt	89 (89)	86 (85.1)	86 (86.9)	91 (91)
RAS wt/BRAF mut	11 (11)	15 (14.9)	11 (11.1)	9(9)
Unknown			2 (2)	
Molecular pathology	*n* = 158		*n* = 156	
dMMR *n* (%)	2 (1.3)		2 (1.3)	
Molecular pathology	*n* = 201		*n* = 199	
BRAFV600E	26 (12.9)		20 (10.3)	
Molecular pathology	*n* = 197		*n* = 195	
PIK3CA	8 (4.1)		17 (9.2)	
Side of primary tumour, *n* (%)				
Left (splenic flexure-rectum)	85 (85)	79 (78.2)	74 (74.7)	70 (70)
Right (transverse colon-caecum)	14 (14)	22 (21.8)	24 (24.2)	30 (30)
Unknown	1 (1)		1 (1)	
Site of primary tumour, *n* (%)				
Colon	57 (57)	59 (58.4)	62 (62.6)	67 (67)
Rectosimgoid	5 (5)	3 (3)	3 (3)	4 (4)
Rectum	37 (37)	39 (38.6)	34 (34.3)	29 (29)
Unknown	1 (1)			
Grading, *n* (%)				
G1	4 (4)	1 (1)		1 (1)
G2	59 (59)	71 (71)	68 (68.7)	63 (63)
G2–3	1 (1)		2 (2)	3 (3)
G3	30 (30)	26 (26)	25 (25.3)	30(30)
GX	6 (6)	3 (3)	4 (4)	3(3)
Number of metastatic sites, No. (%)				
1 site	43 (43)	42 (42)	42 (42.4)	41 (41)
≥2 sites	56 (56)	57 (57)	56 (56.6)	59 (59)
Unknown	1 (1)	1 (1)	1 (1)	
Metastatic sites (selected), No. (%)				
Liver limited	36 (36)	30 (30)	35 (35.4)	32 (32)
Lung	34 (34)	33 (33)	38 (38.4)	42 (42)

Data are number (%).

*FOLFIRI* fluoururacil, folinic acid and irinotecan.

^a^Eastern cooperation of performance status.

The overall response rate was assessable in 353 of 400 patients, while 47 patients were not evaluable as previously reported [20]. The safety profiles in both treatment groups were consistent with the known side-effects of the individual study drugs (Supplementary Appendix. A). Haematological side-effects were numerically higher in younger compared to older patients with regard to Grade-3 (16.9% vs 22.6%) and Grade-4 (4.5% vs 6.0%) toxicities (Supplementary Appendix. A).

### Prognostic impact of age in the *RAS* wild-type population

In the primary analysis of the final *RAS* wild-type population, ORR and PFS were comparable between the treatment groups, but OS was longer in patients treated with FOLFIRI plus cetuximab [20].

In the overall study population (*n* = 400), patients with final *RAS* wild-type tumours aged ≥65 years had a significantly shorter median OS, when compared to younger patients (25.9 months vs 29.3 months, *P* = 0.02, Table 2). In particular, older patients had a markedly shorter survival in the cetuximab arm (27.1 months vs 33.1 months, HR 1.51; 95% CI, 1.10–2.06; *P* = 0.009). In contrast, no age-related effect was observed in the bevacizumab arm (26.0 months vs 25.6 months, HR 1.1; 95% CI, 0.82–1.47; *P* = 0.53, Table 2).

**Table 2:** Primary endpoint ORR and secondary endpoints PFS and OS of FIRE-3 in RAS WT patients grouped in age cohorts and tumour sidedness.

ORR	*n*	RAS WT				*n*	RAS WT right-sided				*n*	RAS WT left-sided			
All	353	FOLFIRI + cet	FOLFIRI + bev	*p*-value		77	FOLFIRI + cet	FOLFIRI + bev	*p*-value		276	FOLFIRI + cet	FOLFIRI + bev	*p*-value	
Patients <65 years	181	*n* = 84 ORR 76.2%	*n* = 97 ORR 62.9%	0.076		32	*n* = 11 ORR 63.6%	*n* = 21 ORR 52.4%	0.71		149	*n* = 73 ORR 78.1 %	*n* = 76 ORR 65.8 %	0.11	
Patients ≥65 years	172	*n* = 86 ORR 77.9%	*n* = 86 ORR 66.3%	0.13		45	*n* = 19 ORR 68.4%	*n* = 26 57.7%	0.54		127	*n* = 67 ORR 80.6%	*n* = 60 ORR 70.0 %	0.22	
**PFS (months, 95% CI)**		**RAS WT**					**RAS WT right-sided**					**RAS WT left-sided**			
All	400	FOLFIRI + cet	FOLFIRI + bev	*p*-value	HR	90	FOLFIRI + cet	FOLFIRI + bev	*p*-value	HR	308	FOLFIRI + cet	FOLFIRI + bev	*p*-value	HR
Patients <65 years	201	11.1 (9.3–13)	10.2 (8.7–11.7)	0.46	0.9 (0.67–1.2)	36	7.2 (3.5–11)	7.2 (4.3–10.0)	0.19	1.6 (0.78–3.32)	164	12 (9.5–14.6)	10.3 (8.8–11.9)	0.3	0.85 (0.62–1.16)
Patients ≥65 years	199	9.9 (8.4–11.4)	11.0 (9.6–12.6)	0.82	1.03 (0.7–1.4)	54	8.0 (6.1–9.8)	9.3 (6.6–12.1)	0.44	1.2 (0.71–2.17)	144	10.4 (9.4–11.4)	11.1 (9.6–12.6)	0.94	0.99 (0.70–1.4)
**OS (months, 95% CI)**		**RAS WT**					**RAS WT right-sided**					**RAS WT left-sided**			
All	400	FOLFIRI + cet	FOLFIRI + bev	*p*-value	HR	90	FOLFIRI + cet	FOLFIRI + bev	*p*-value	HR	308	FOLFIRI + cet	FOLFIRI + bev	*p*-value	HR
Patients <65 years	201	33.1 (26.9–39.2)	25.6 (19.1–32.2)	0.012	0.67 (0.5–0.91)	36	21.9 (14.6–29.2)	16.4 (8.3–24.7)	0.31	0.68 (0.32–1.44)	164	38.2 (30.9–45.4)	28.2 (22.7–33.7)	0.006	0.62 (0.44–0.88)
Patients ≥65 years	199	27.1 (18.9–35.3)	26.0 (23.0–28.7)	0.46	0.89 (0.66–1.2)	54	16.6 (11.6–20.5)	23.6 (19.3–27.9)	0.87	1.1 (0.6–1.87)	144	33.2 (21.3–45.1)	27.5 (24.0-31-0)	0.38	0.86 (0.60–1.2)

For survival times Kaplan–Meier estimation medians and 95% CIs are given.

*FOLFIRI* fluorouracil, leucovorin and irinotecan, *cet C*etuximab, *bev* Bevacizumab, *HR* hazard ratio, *OS* overall survival, *PFS* progression-free survival, *wt* wild-type.

### Impact of age on treatment efficacy of cetuximab and bevacizumab in left- and right-sided primary tumours

**Fig. 1:**
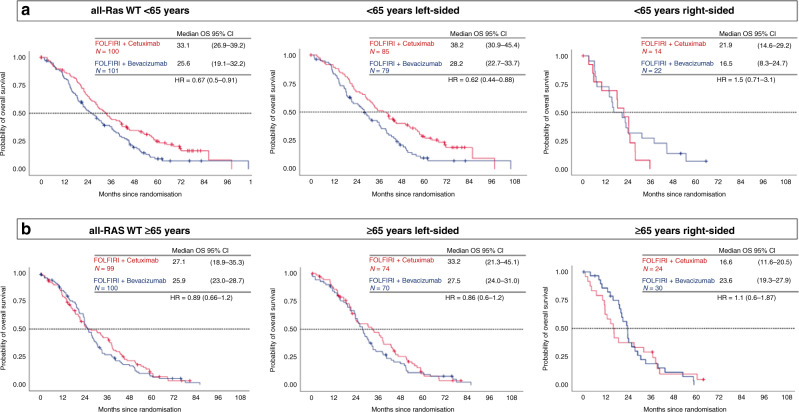
Age correlated overall survival according to treatment group in the RAS wild-type population. For survival times Kaplan–Meier estimation medians and 95% CIs are given. FOLFIRI fluorouracil, leucovorin, and irinotecan, HR hazard ratio, OS median overall survival, wt wild-type.

Considering all tumour localisations together, the comparison of OS by treatment arm clearly demonstrated that FOLFIRI plus cetuximab was markedly superior to FOLFIRI plus bevacizumab in patients aged ≤65 years (OS 33.1 months vs 25.6 months; HR 0.67; 95% CI, 0.5–0.91; *P* = 0.012, Fig. 1a). Within the older patients, no significant difference in efficacy between cetuximab- and bevacizumab-treated patients could be observed (27.1 vs. 26.0 months; HR 0.89; 95% CI, 0.66–1.2; *P* = 0.46, Table 2). The analysis of ORR in younger patients showed a markedly greater benefit from cetuximab compared to bevacizumab (76.2% vs 62.9%, *P* = 0.076). In older patients ORR was only numerically greater in the cetuximab arm (77.9% vs 66.3%, *P* = 0.13) and this effect did not reach the level of statistical significance (Table 2). For further clarification, the age subgroup analysis was extended to sidedness (left versus right) of colorectal cancer. In the overall study population, patients with left-sided primary tumours lived longer compared to patients with right-sided primary tumours (30.8 months versus 21.2 months, *P* < 0.01). Only 90 patients with right-sided primary tumours were evaluated in the present analysis (36 patients aged <65 years and 54 patients aged ≥65 years, Table 2).

In the left-sided, *RAS* wild-type, colorectal cancer population <65 years, OS was significantly longer in cetuximab- compared to bevacizumab-treated patients (38.2 months vs. 28.2 months; HR 0.62, 95% CI 0.44–0.88; *P* = 0.006, Fig. 1). PFS and ORR were only numerically greater in the cetuximab arm (PFS: 12 vs 10.3 months, *P* = 0.3; 78.1% vs 65.8%, *P* = 0.11, Table 2).

In patients with right-sided, *RAS* wild-type tumours <65 years, the difference between treatment groups lost the level of significance and cetuximab led only numerically to superiority (21.9 vs 16.4, *P* = 0.31). PFS and ORR were comparable between treatment groups (PFS 7.2 vs 7.2, *P* = 0.19; 63.6% vs 52.4%, *P* = 0.71, Table 2).

For patients older than 65 years with left-sided CRC, the increase of median OS in cetuximab- compared to bevacizumab-treated patients was just numerically evident (33.2 vs 27.5 months, HR 0.86; 95% CI, 0.6–1.2; *P* = 0.38, Table 2) and also ORR did not reach level of significance (80.6% vs 70.0%, *P* = 0.22, Table 2). PFS did not significantly differ between the cetuximab group and the bevacizumab group (Table 2). Importantly, older patients with right-sided primaries did not appear to benefit from cetuximab as compared to bevacizumab with regard to median OS (16.6 months vs 23.6 months, HR 1.1; 95% CI, 0.6–1.87; *P* = 0.87, Fig. 1b, Table 2). PFS and ORR resembled between treatment groups (PFS 8.0 vs 9.3, *P* = 0.44; 68.4% vs 57.7%, *P* = 0.54, Table 2). The median OS outcomes in older patients contrast those in younger patients (OS 21.9 months versus 16.4 months, HR 0.68; 95% CI, 0.32–1.44; *P* = 0.31, Table 2).

### Potential drivers accounting for differences in efficacy

To rule out the possibility that less therapy may account for the shorter survival of older patients, the number of treatment cycles within the study as well as further lines of therapy were examined.

Older patients received comparable numbers of treatment cycles with FOLFIRI/cetuximab vs FOLFIRI/bevacizumab (12.7 vs 12.9) compared to younger patients (13.1 vs 14.5). Also the percentage of patients receiving second- and third-line chemotherapy was not significantly different between younger and older patients (Table 3).

**Table 3:** Further line treatments and early tumour changes according to age, treatment arm and tumour sidedness.

Characteristic	Age analysis set							
Age	<65 years				≥65 years			
* n* (%)	201 (50.2)				199 (49.8)			
Treatment arm	**FOLFIRI** + **cetuximab**		**FOLFIRI** + **bevacizumab**		**FOLFIRI** + **cetuximab**		**FOLFIRI** + **bevacizumab**	
* n*	100		101		99		100	
Side of primary tumour	**Left**	**Right**	**Left**	**Right**	**Left**	**Right**	**Left**	**Right**
Number of cycles completed, No.	13.1		14.5		12.7		12.9	
Lines of therapy administered, *n* (%)								
Second line	74 (74)		75 (74.3)		75 (75.8)		74 (74)	
Third line	53 (53)		55 (54.5)		52 (52.5)		46 (46)	
Secondary resection rate, *n* (%)	51 (25.4)				35 (17.6)			
	26 (26)		25 (24.8)		13 (13.1)		22 (22)	
	*n* = 85	*n* = 14	*n* = 79	*n* = 22	*n* = 74	*n* = 24	*n* = 70	*n* = 30
	24 (28.2)	2 (14.3)	22 (27.8)	3 (13.6)	11 (14.9)	2 (8.3)	18 (25.7)	4 (13.3)
Early tumour shrinkage (ETS), *n* (%)	*n* = 76		*n* = 92		*n* = 88		*n* = 83	
	51 (67.1)		47 (51.1)		60 (68.2)		38 (45.8)	
	*n* = 67	*n* = 8	*n* = 72	*n* = 20	*n* = 65	*N* = 23	*n* = 62	*n* = 21
	45 (67.2)	6 (75)	38 (52.8)	9 (45)	49 (75.4)	11 (47.8)	29 (46.8)	9 (42.9)
Depth of response (DPR)	*n* = 73		*n* = 91		*n* = 84		*n* = 82	
	−42		−31		−27.8		−18.2	
	*n* = 64	*n* = 8	*n* = 71	*n* = 20	*n* = 62	*n* = 22	*n* = 70	*n* = 20
	−42.4	−43.1	−34.2	−19.6 (36.5)	−33.8	−10.8	−21	−9.7
Grade of acneiform exanthema 2–3, *n* (%)	53 (53)		1 (1)		48 (48.5)		3 (3)	
	*n* = 85	*n* = 14	*n* = 79	*n* = 22	*n* = 74	*n* = 24	*n* = 70	*n* = 30
	49 (57.6)	4 (28.6)	1 (1.3)	0	38 (51.4)	10 (41.7)	3 (4.3)	0

Data are number (%).

*FOLFIRI* fluoururacil, folinic acid and irinotecan.

To account for potential drivers of the inferior outcomes of older patients, further exploratory analyses were conducted. Exploration of early tumour shrinkage (ETS) and depth of response (DpR) according to age groups showed no statistical difference (Table 3). In addition, patients were classified according to clinically significant cetuximab-induced skin toxicity, but no significant difference in severity was observed in older patients who received cetuximab (Table 3).

**Fig. 2:**
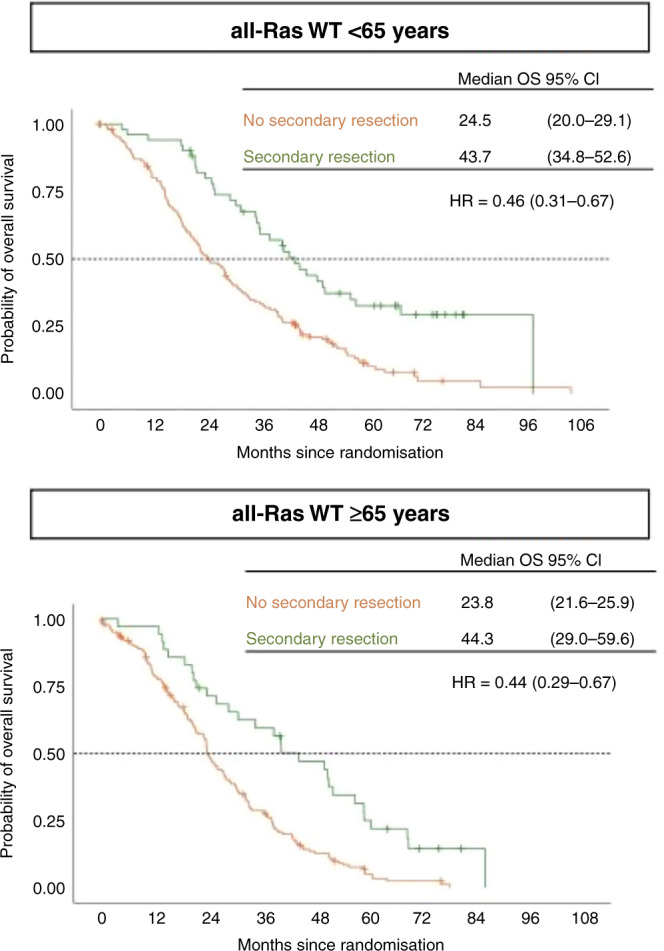
Age correlated overall survival according to secondary resection in the RAS wild-type population. For survival times Kaplan–Meier estimation medians and 95% CIs are given. OS median overall survival, wt wild-type.

Secondary resection rates were numerically higher in younger patients (25.4% vs. 17.6%, *P* = 0.068) than in older patients. Mainly in the FOLFIRI + cetuximab arm, older patients underwent less likely resection (13.1% vs. 26%; *P* = 0.02, Table 3). Both, older and younger patients with resectable disease that actually underwent resection had superior overall survival (44.3 months vs 23.8 months, HR 0.44; 95% CI, 0.29–0.67; *P* < 0.005 and for younger patients 43.7 months vs 24.5 months, HR 0.46; 95% CI, 0.31–0.67; *P* < 0.005, Fig. 2), notably patients with left-sided tumours (data not shown). In patients who underwent resection overall survival between younger and older patients was similar (43.7 months vs 44.3 months, HR 1.3; 95% CI, 0.8–1.22; *P* = 0.29).

## Discussion

The present analysis of the FIRE-3 trial was performed as a post-hoc analysis of patients with *RAS* wild-type mCRC grouped in two age cohorts. Two questions were investigated: Firstly, to what extent overall survival depends on age; secondly, which targeted therapy, as an addition to first-line treatment with FOLFIRI, should be preferred in relation to age and sidedness of tumour. In this context, it is relevant to point out that patients included in FIRE-3 were deemed fit for combination chemotherapy plus targeted therapy independent of age. Hence, the comparison of an older versus a younger age group in this study does not necessarily reflect the comparison of a frail versus a fit subgroup of patients.

Comparing different age cohorts in the overall study population of FIRE-3, OS was significantly shorter in the age cohort ≥65 years as compared to younger patients. This effect is likely multifactorial. Firstly, it could be attributed to a higher proportion of right-sided tumours in older patients (Table 1). As expected, older patients (≥65 years) in FIRE-3 showed a higher proportion of right-sided tumours than younger patients. In accordance with the published literature, right-sided primary tumour location was associated with markedly shorter OS compared to left-sided primary tumour location when considering the overall study population. This effect was observed in the younger as well as in the older patient cohort. Secondly, younger patients showed a trend toward a higher secondary resection rate. Secondary resection is associated with a better OS [23]. To assess, if older patients profit as same as younger patients from secondary resection, OS was compared between younger and older resected patients and showed no difference. So, in the FIRE-3 population, secondary resection in older patients was safely performed and resulted in better OS.

Evaluation of the study population according to targeted therapy demonstrated that significantly shorter survival in older versus younger patients was observed only in the cetuximab arm (HR 1.5, *P* < 0.01), but not in bevacizumab-treated patients (HR 1.1, *P* = 0.53) of FIRE-3. This finding may point to an age-related effect of anti-EGFR directed therapy. It has not been reported previously and thus needs confirmation. A similar observation was made by Garcia-Alfonso et al. 2021 but in the meta-analysis, factors like worse ECOG and a lower percentage of active treatment after first-line therapy have contributed to the shorter OS of older patients [24]. In the present analysis, there were no differences in ECOG or lower percentage of second or third-line therapy but older patients treated with cetuximab had a lower secondary resection rate compared to older patients treated with bevacizumab (Table 3).

Previous reports have demonstrated that combination chemotherapy may improve PFS and OS in younger as well as in older patients [18, 25, 26]. In the present study, combination chemotherapy was used as a backbone treatment with the expectation that tolerability and efficacy would be acceptable and comparable through different age groups. The present evaluation asks the question if this assumption also holds for targeted therapy with specific regard to anti-EGFR- versus anti-VEGF-directed therapy.

Previous studies support the notion that the addition of bevacizumab to FP significantly improved PFS in older patients, while the effect on OS remains less clear [12–16]. There is less evidence on cetuximab-based combination therapy in first-line treatment of older mCRC patients. A combination of cetuximab with single-agent chemotherapy was shown to be more effective in *KRAS* WT mCRC patients [27]. In addition, non-interventional and retrospective investigations revealed the efficacy of cetuximab plus irinotecan in older patients with pre-treated *KRAS* WT mCRC and had a similar safety profile compared to younger patients [28, 29]. However, in the PRIME study, the addition of Panitumumab to first-line treatment with FOLFOX-4 showed OS benefit in the cohort of RAS wild-type patients <65 years but not for older patients [30].

In the present investigation of older patients (≥65 years) with *RAS* WT mCRC, there were no significant differences between cetuximab- or bevacizumab-based therapy with regard to ORR, PFS or OS. This result is in clear contrast to younger patients (<65 years) as well as to the unselected population where FOLFIRI plus cetuximab led to a significantly longer OS when compared to FOLFIRI plus bevacizumab [20]. Translating these results into clinical practice would mean that in older patients with *RAS* WT mCRC there is no favoured targeted therapy, since both anti-EGFR- and anti-VEGF-directed treatment yields comparable results. Due to a major survival advantage (HR 0.67, *P* = 0.012), there is, however, a strong recommendation to use first-line cetuximab in younger patients.

Primary tumour sidedness is not only an important prognostic factor in metastatic colorectal cancer, but it is also predictive as it affects the response to anti-EGFR-directed therapies. Thus it was shown that cetuximab-based therapy resulted in better outcomes in patients with left- compared to right-sided tumours *KRAS* wild-type mCRC [2, 31, 32].

Good evidence exists that patients with right-sided mCRC do either not benefit or even derive a disadvantage from anti-EGFR-directed treatment [33, 34].

A strong and statistically significant superiority of cetuximab over bevacizumab was specifically shown in patients with left-sided mCRC aged <65 years. Older patients with left-sided primary tumours treated with cetuximab had a not significantly prolonged OS when compared to bevacizumab. In contrast, in patients with right-sided primaries no benefit from cetuximab was observed independent of age.

Potential limitations of our study include its retrospective nature as well as the limited patient numbers in analysed subgroups. This issue becomes relevant specifically with regard to the subgroup of right-sided cancers, which is notably smaller than left-sided ones [3, 8, 35].

For the purpose of comparing equally sized groups, the present study elected to choose an age cut-off of 65 years. This had several reasons. Firstly, eligible patients in FIRE-3 were aged 18–75 years so subdivision of the study population at a cut-off of 65 years resulted in nearly equally sized patient cohorts. While patients aged ≥65 years clearly do not represent an elderly population per se, there is no widely accepted cut-off that defines the so-called elderly population in patients with metastatic colorectal cancer. Nevertheless, the present observations must be considered mainly as hypothesis-generating and clearly require confirmation by prospective studies.

Treatment recommendations for older patients with metastatic disease should focus not only on the molecular biology of the tumour but must also allow for the development of personalised multidisciplinary strategies, considering fitness, comorbidities and expectations of the patient as well as anticipated side-effects of antineoplastic therapy.

## Conclusions

The results presented here suggest that older patients have a shorter OS despite intensive treatment. This observation could be explained by the overrepresentation of right-sided tumours in elderly patients. Furthermore, in our cohort, older patients were less likely to undergo secondary resection. However, secondary resection was equally beneficial in older and younger patients.

According to our analyses, for older patients, no favoured targeted therapy emerged as both anti-EGFR- and anti-VEGF-directed treatment yielded comparable results. These findings could be taken into account in the multidisciplinary management of older patients suffering from *RAS* wild-type colorectal cancer.

## Supplementary information

Supplementary Appendix

aj-checklist

## Data Availability

All authors had access to the data published in this paper. The datasets used and analysed during the current study are available from the corresponding author on reasonable request.
