# Supplementary material for: Efficacy of FOLFIRI plus cetuximab vs FOLFIRI plus bevacizumab in 1st-line treatment of older patients with RAS wild-type metastatic colorectal cancer: an analysis of the randomised trial FIRE-3
Source: Br J Cancer. 2022 May 30;127(5):836–43. doi: 10.1038/s41416-022-01854-y (PMC9427779; doi:10.1038/s41416-022-01854-y)
Supplement: Supplementary file 1 — Supplementary Appendix [file 41416_2022_1854_MOESM1_ESM.docx]

**Supplementary Appendix. A**

**TABLE 1: Number of grade 3 to 5 adverse events of interest according to age subgroups of FIRE-3 in RAS wild-type patients.**

| Adverse Event | ˂ 65 years  n=201 (50.2 %) | | | ≥65 years  n=199 (49.8%) | | |
| --- | --- | --- | --- | --- | --- | --- |
| CTCAE¹ | **Grade 3** | **Grade 4** | **Grade 5** | **Grade 3** | **Grade 4** | **Grade 5** |
| Hematotoxicity | 34 | 9 |  | 45 | 11 | 1 |
| Infection | 6 | 1 |  | 9 |  | 2 |
| Mucositis | 7 | 1 |  | 10 | 1 | 1 |
| Electrolyte imbalance | 9 |  |  | 9 | 3 |  |
| Fatigue |  |  |  | 3 |  |  |
| Pain | 2 |  |  | 2 |  |  |
| Allergic reaction | 4 | 1 |  | 3 | 1 |  |
| Thromboembolia | 4 | 5 |  | 4 | 3 | 7 |
| Nausea | 6 |  |  | 9 |  |  |
| Vomiting | 4 |  |  | 6 |  |  |
| Skin disorder | 2 |  |  | 6 | 1 |  |
| Hand-foot-syndrome | 6 |  |  | 5 |  |  |
| Liver toxicity | 1 |  |  | 6 |  |  |
| Nephrotoxicity | 1 |  |  | 2 |  |  |
| Rash | 26 |  |  | 17 |  |  |
| Pruritus | 2 |  |  | 1 |  |  |
| Nail changes | 6 |  |  | 8 |  |  |
| Diarrhoea | 19 | 2 |  | 23 | 4 |  |
| Cardiac Toxicity | 1 |  |  | 4 | 1 | 1 |
| Obstipation |  |  |  | 1 |  |  |
| Neurotoxicity | 1 |  |  | 1 |  |  |
| Hypertension | 3 |  |  | 7 |  |  |
| Bleeding | 1 |  |  |  |  |  |

**Legend:** Adverse event (AE) derived from case report forms. All events were recorded as related to study treatment. Adverse events were documented according to The National Cancer Institute Common Terminology Criteria for Adverse Events (v4.0). Numbers represent absolute cases. ¹CTCAE= Common Terminology Criteria for Adverse Events.
